# Theoretical Investigation of Electric Polarizability in Porphyrin–Zinc and Porphyrin–Zinc–Thiazole Complexes Using Small Property-Oriented Basis Sets

**DOI:** 10.3390/ijms252011044

**Published:** 2024-10-14

**Authors:** Arkadiusz Kuziemski, Krzysztof Z. Łączkowski, Angelika Baranowska-Łączkowska

**Affiliations:** 1 Faculty of Physics, Kazimierz Wielki University, Powstańców Wielkopolskich 2, 85-090 Bydgoszcz, Poland; arek.kuziemski@gmail.com; 2 Department of Chemical Technology and Pharmaceuticals, Faculty of Pharmacy, Collegium Medicum, Nicolaus Copernicus University, Jurasza 2, 85-089 Bydgoszcz, Poland; krzysztof.laczkowski@cm.umk.pl

**Keywords:** transition metals, porphyrin–zinc–thiazole complex, basis set, electric polarizability, DFT calculation

## Abstract

Porphyrin complexes are of great importance due to their possible applications as sensors, solar cells and photocatalysts, as well as their ability to bind additional ligands. A valuable source of knowledge on their nature is their electric properties, which can be evaluated employing density functional theory (DFT) methods, supporting the experimental research. The present work aims at the application of small property-oriented basis sets in calculation of electric properties in transition metals, their oxides and test coordination complexes. Firstly, the existing polarized ZPol basis set for the first-row transition metals is modified in order to improve atomic polarizability results. For this purpose, optimization of the f-type polarization function exponent is carried out with respect to the value of average atomic polarizability of investigated metals. Next, both the original and the modified basis sets are employed in finite field CCSD(T) calculation of transition metal oxides’ dipole moments, as well as DFT calculation of polarizabilities in porphyrin–zinc and porphyrin–zinc–thiazole complexes. The obtained results show that the ZPol and ZPol-A basis sets can be successfully employed in the calculation of linear electric properties in large systems. The optimization procedure used in the present work can be employed for other source basis sets and elements, leading to new efficient polarized basis sets.

## 1. Introduction

Transition metals are chemical elements localized in the d-block of the periodic table. They form numerous compounds, among them coordination complexes, often exhibiting different oxidation states. Found in the active centers of many enzymes, transition metals are essential to basic biological processes in human and other living organisms. They also have wide applications, e.g., as catalysts for chemical reactions and medicines [1,2,3,4]. In particular, porphyrin complexes with transition metals are of great importance due to their possible applications: metalloporphyrins are used as sensors, solar cells and photocatalysts, and their ability to bind additional ligands determines their role in enzymes [5,6,7,8]. Metalloporphyrins can also have promising antifungal and anticancer activities [9]. The present study investigates the properties of porphyrin–zinc and porphyrin–zinc–thiazole complexes. Thiazole moiety is present in many compounds possessing anticancer, antimicrobial and antioxidant properties [10,11,12,13,14,15], and thus complexes of thiazole and metalloporphyrins are of large importance due to their potential medicinal applications.

The research on metalloporphyrins is often focused on better understanding of the nature of these systems. One of the valuable sources of information on these systems is their electric properties; however, experimental investigation is often difficult to carry out and encounters limitations. Fortunately, experimental results can be estimated or verified using available theoretical tools. While, in some applications, calculation based on molecular mechanics or molecular dynamics is sufficient, there is a wide number of properties that need more accurate computational methods based on quantum mechanics. Examples of theoretical studies carried out for metalloporphyrins can be found in the literature [16,17,18,19,20,21,22,23,24,25].

Theoretical investigation of electric polarizability has to be carried out using methods based on quantum mechanics. Unfortunately, these methods are characterized by a large computational cost. In general, this cost increases fast with the basis set size, that is the number N of functions used to model atomic and molecular orbitals. E.g., coupled cluster (CC) theory-based approaches scale with the number of basis set functions N being N^6^ (coupled cluster singles and doubles method, CCSD) or even N^7^ (coupled cluster singles and doubles method with noniterative perturbational treatment of triple excitations, CCSD(T)). Thus, with the increase of investigated system size, the calculations soon become prohibitive. A way around is the use of methods which scale better with the number of basis set functions, e.g., methods based on density functional theory (DFT) or scaling as N^3^ or—in the case of hybrid exchange–correlation functionals—N^4^. DFT methods are preferred in the case of investigations of large organic systems when a quantum mechanical approach becomes necessary. Nevertheless, even such lower-cost methods imply large computing costs for complexes built of a dozen or more of the so-called heavy atoms if a large basis set is used.

Decreasing the computing cost is possible through the employment of smaller basis sets, containing a smaller number of functions per atom. In general, however, this means a less accurate description of the system, and thus can lead to a significant deterioration of results, as, in general, the larger and more flexible the basis set used, the more accurate the description of orbitals that can be obtained. It is thus important to carefully select both the method of calculation and the basis set when looking for a balance between the cost of quantum mechanical calculations and their accuracy.

The above-mentioned problem can be solved in two ways. On the one hand, efforts can be made to obtain algorithms that scale better with the number of functions. On the other hand, reduced-size but flexible enough basis sets can be developed. In the present work, we focus on the latter approach, employing in our calculations a basis set belonging to the Pol sets family [26,27,28,29,30,31,32,33]. The compact ZPol basis set used in the present work was originally developed for calculations of molecular linear electric properties [29,30,31]. It was designed based on conclusions drawn from the harmonic oscillator model in time-dependent external electric fields, see works [28,29,30,31] for details. The method of generation of polarization functions in the ZPol set makes it a small but potent tool, providing fairly accurate results for linear electrical properties. However, to our knowledge the ZPol set published for the first-row transition metals has not yet been employed in calculations for transition metal organic complexes.

The aim of this work is to employ the electric property-oriented ZPol basis set for the first time in calculations of the electric polarizabilities of the porphyrin–zinc and porphyrin–zinc–thiazole complexes. For that purpose, we try to improve the ZPol basis set quality through optimization of orbital exponents for the first order polarization functions. Instead of the contracted f-type function from the original ZPol set, we use a single uncontracted function. Optimal values of the orbital exponents are chosen to be those minimizing errors in atomic polarizability values with respect to the reference values by Stiehler and Hinze [34]. Next, we test the resulting basis set in the finite field CCSD(T) calculations of the dipole moments of the first-row transition metal oxides. The results are compared with the available theoretical data [35,36]. Moreover, we calculate dipole moments of oxides using the aug-cc-pVQZ basis set [37] to provide additional reference results. Finally, we employ both the original and the modified ZPol basis sets for zinc atoms in DFT calculations of the molecular electric polarizabilities of the porphyrin–zinc and porphyrin–zinc–thiazole complexes. The 6-311G** basis set is used for all other atoms [38]. Due to the lack of experimental reference values, we evaluate the corresponding polarizabilities using the aug-cc-pVXZ basis set on the zinc atom, as well as the def2-SVPD and def2-TZVPD basis sets of Rappoport and Furche [39,40]. The Becke 3-parameter Lee–Yang–Parr (B3LYP) hybrid exchange–correlation functional [41,42,43] is employed.

## 2. Results and Discussion

The optimal modified basis set is referred to as the ZPol-A basis set in the following, and the values of its first-order f-type polarization function exponents are listed in Table 1.

A summary of the atomic polarizability results obtained within the ZPol-A basis set is presented in Table 2, together with the values reported in [31] for the original ZPol set and reference values from [34].

The largest discrepancy between the property-oriented basis sets values and reference results of Stiehler and Hinze is observed for chromium polarizability (the errors calculated with respect to the reference values equal 5.9% and 5.4% for ZPol and ZPol-A, respectively) and copper (errors of 4.8% and 4.6% for ZPol and ZPol-A, respectively). For the remaining investigated transition metals, the errors do not exceed 2%, which is an optimistic result for such compact basis sets. For all first-row transition metals and all polarizability components, the ZPol-A set outperforms the ZPol set, yielding results about 0.3–0.6% closer to the reference values. The largest improvement in accuracy is observed for titanium (average polarizability 0.66% closer to the reference) and vanadium (average polarizability 0.57% closer to the reference).

The electric dipole moments of the investigated oxides calculated using the ZPol-A basis set are presented in Table 3 together with the corresponding ZPol values reported in [31]. They are compared to the aug-cc-pVQZ values obtained within the present study, as well as reference data from [35,36]. The results obtained using the aug-cc-pVQZ basis set are of particular importance in the cases of FeO, CoO and NiO, where accurate literature reference data are not available.

Analysis of Table 3 reveals that the agreement of both ZPol and ZPol-A electric dipole moment values with the reference data is very good. The root mean square error (RMSE) values calculated for the seven investigated oxides with respect to the available literature data [35,36] is slightly smaller in the case of the original ZPol basis set. The ZPol-A basis set competes with the original ZPol set for ScO and ZnO. Considering the aug-cc-pVQZ reference results, the RMSE values are identical for both basis sets, with the ZPol-A set outperforming the original ZPol set for ScO, CrO and CoO, and with the results obtained in both these basis sets for ZnO being almost identical.

**Figure 1:**
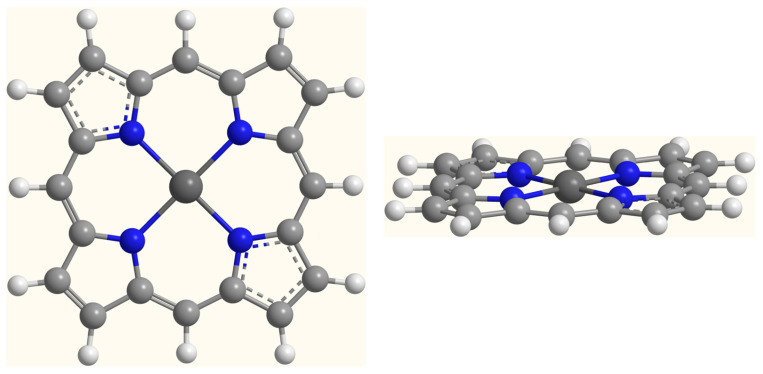
The B3LYP/6-311G**/LanL2DZ optimized structure of the porphyrin–zinc complex. White, light grey, dark grey and blue spheres represent hydrogen, carbon, zinc and nitrogen atoms, respectively. Values of geometrical parameters are reported in Table S1 of Supporting Information.

**Figure 2:**
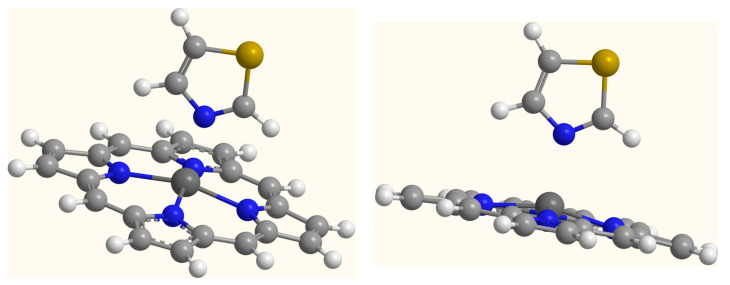
The B3LYP/6-311G**/LanL2DZ optimized structure of the porphyrin–zinc–thiazol complex. White, light grey, dark grey, yellow and blue spheres represent hydrogen, carbon, zinc, sulfur and nitrogen atoms, respectively. Values of geometrical parameters are reported in Table S2 of Supporting Information.

Optimized geometrical parameters of the porphyrin–zinc and the porphyrin–zinc–thiazole complexes are presented in Tables S1 and S2 of the Supporting Information. Figure 1 and Figure 2 depict the B3LYP/6-311G**/LanL2DZ optimized structures of the investigated systems. The porphyrin–zinc complex is planar, with four nitrogen atoms coordinating around the zinc atom. A penta-coordinated arrangement is observed for the porphyrin–zinc–thiazole complex, with thiazole being the axial ligand. Due to the interaction between the zinc atom and the nitrogen atom of the thiazole molecule, the zinc atom does not lie in the porphyrin plane.

Polarizability values obtained for the tested porphyrin complexes are listed in Table 4. In the following, we assume the results obtained using the largest basis set on the zinc atom, that is the aug-cc-pVQZ set, to be the reference, i.e., 340.0 au for the porphyrin–zinc and 386.1 au for the porphyrin–zinc–thiazole complex. Due to the differences observed between the values obtained in the 6-311G**/aug-cc-pVQZ and 6-311G**/aug-cc-pVTZ basis sets, it can be concluded that the reference values could still somewhat change when moving to the larger or more diffuse basis sets of Dunning.

The result obtained with the ZPol or ZPol-A basis sets placed on the zinc atom leads to a very good agreement with the reference results for both investigated complexes. The differences are in the order of 2.1 au (ZPol set) and 2.3 au (ZPol-A set) for the porphyrin–zinc complex, and 3.5 au (ZPol set) and 3.7 au (ZPol-A set) for the porphyrin–zinc–thiazole complex. This corresponds to an error of 0.6–0.7% in the case of the porphyrin–zinc complex, and 0.9–1.0% for porphyrin–zinc–thiazole complex. From the point of view of molecular applications of the investigated systems, these errors are acceptable. It is worth mentioning that the ZPol and ZPol-A basis sets contain 43 contracted functions per zinc atom, while the aug-cc-pVDZ, aug-cc-pVTZ and aug-cc-pVQZ sets have 59, 93 and 140 functions per zinc atom, respectively. The reduction in computational cost is therefore significant, especially for systems containing a larger number of transition metal atoms. The def2-SVPD and def2-TZVPD sets also yield promising results. The def-SVPD set is exceptionally small with its 34 basis functions. Placed on the zinc atoms, it gives errors of 2.9 au (0.8%) and 4.0 au (1.0%) for the porphyrin–zinc and porphyrin–zinc–thiazole complexes, respectively. The def2-TZVPD set contains 51 functions and leads to errors of 1.6 au (0.5%) and 2.2 au (0.6%).

## 3. Materials and Methods

In order to modify the ZPol basis set, values of the orbital exponents of f-type polarization functions were optimized. In the original work [31], the contracted f-type function was obtained based on the conclusions from the harmonic oscillator in time-dependent electric fields. Specifically, polarization functions for an angular momentum quantum number equaling *l* + 1 were generated from the—already present in the initial basis set—functions corresponding to an angular momentum quantum number equaling *l*, through simple scaling of their contraction coefficients and reduction of the linear combination to the most diffuse primitive functions. Thus, the polarization functions in the ZPol basis set had the same values of orbital exponents as those already present in the initial set for the functions corresponding to an angular momentum quantum number equaling *l*. Presently, a single uncontracted f-type Gaussian-type function was used, and the value of its orbital exponent was chosen based on the results of calculations of static atomic polarizability carried out within the finite field approximation and the restricted open-shell Hartree-Fock (ROHF) method, assuming C_2v_ symmetry. The external electric field strength was taken as being equal to ±0.001 au. For chromium, manganese, copper and zinc the only possible value of the $M_{L}$ quantum number is 0, and thus a single polarizability value was calculated. For the other investigated elements, L≠0, so polarizability calculations were carried out for all possible components, and the average polarizability value was used as a reference. For titanium, vanadium, cobalt and nickel, mixing of the states with $M_{L}=1$ and $M_{L}=3$ was observed, and thus the corresponding polarizability components were calculated from the equation [30]

(1) $${\alpha (M}_{L})=(L^{2}-M_{L}^{2})A+(L^{2}-2L+1-M_{L}^{2})C$$

with L being the total orbital angular momentum quantum number, while A and C are constants determined from polarizability values obtained for the remaining components.

The following modification of the ZPol basis set was carried out: instead of the original contracted f-type function, a single uncontracted f-type polarization function was used, with different orbital exponent values. Its value was taken to be from 0.01 to 0.1 with a step of 0.01, and from 0.1 to 10.0 with a step of 0.1. These modified basis sets were used in the finite field atomic polarizability calculations, assuming the external electric field value to be equal to 0.001, and the results were compared to the available literature results [34]. Next, in the vicinity of the exponent values yielding results closest to the reference, analogous iterations were performed using smaller steps, until the obtained polarizability value did not change. The resulting optimal modified ZPol basis set is referred to as ZPol-A. It was next employed in the calculations of the dipole moments of the studied transition metal oxides, as well as the polarizability of the porphyrin–zinc and the porphyrin–zinc–thiazole complexes.

The electric dipole moments of the oxides were calculated using the finite field approximation and the CCSD(T) method. The original ZPol set was used on the oxide atoms. Intermolecular distances adopted from [35] were used, that is R_ScO_ = 3.174 au, R_TiO_ = 3.077 au, R_VO_ = 3.027 au, R_CrO_ = 3.087 au, R_MnO_ = 3.146 au, R_FeO_ = 3.041 au, R_CoO_ = 3.063 au, R_NiO_ = 3.073 au, R_CuO_ = 3.347 au and R_ZnO_ = 3.248 au. To better evaluate the performance of the ZPol and ZPol-A basis sets, dipole moments were additionally calculated using the aug-cc-pVQZ basis set.

In order to evaluate average static polarizabilities of porphyrin–zinc and porphyrin–zinc–thiazole complexes, first the optimization of geometrical parameters was carried out using the DFT/B3LYP [41,42,43] approximation, the LanL2DZ basis set on the zinc atom [44] and the 6-311G** basis set on the remaining atoms. Next, average polarizabilities were calculated for the optimal geometry of the complexes, using the DFT/B3LYP method and the ZPol, ZPol-A, def2-SVPD, def2-TZVPD and aug-cc-pVXZ (X = D, T, Q) basis sets on the zinc atom, and the 6-311G** basis set on the remaining atoms.

All finite field calculations for atoms and oxides were carried out using the MOLCAS 7.8 program [45], while geometry optimization and polarizability calculations for the porphyrin–zinc and porphyrin–zinc–thiazole complexes were performed using the Gaussian 09 program [46].

## 4. Conclusions

In the present work, the property-oriented ZPol basis set was successfully modified to yield the atomic polarizabilities of the first-row transition metals with better accuracy. The finite field ROHF results obtained for the first-row transition metal atoms show that the ZPol-A set leads to atomic polarizability results in better agreement with accurate reference data than does the standard ZPol set, while not causing any increase in the computing cost.

Next, the ZPol-A basis set was employed in finite field CCSD(T) calculation of electric dipole moments in first-row transition metal oxides. The performance of the ZPol and ZPol-A basis sets in the calculation of electric dipole moments was very good. Both sets lead to small values of RMSE calculated with respect to the literature data [35,36], and with respect to the aug-cc-pVQZ results of the present work. Calculations performed for transition metal oxides confirmed that the ZPol-A set can be safely used in the evaluation of linear electric properties.

Finally, both basis sets were successfully employed in DFT/B3LYP calculation of the electric polarizability of porphyrin–zinc and porphyrin–zinc–thiazole complexes. Their use on the zinc atom, together with the 6-311G** basis set on other atoms, allowed us to obtain results very close to the reference values reported in this work. The differences between the results obtained using the ZPol and ZPol-A sets on the zinc atom were not large, allowing us to conclude that both sets are promising tools for calculations of electric properties in transition metal complexes. It has to be mentioned that the B3LYP functional does not include long-range corrections, and as such can lead to inaccurate results of response properties. However, this shortcoming of the B3LYP calculations becomes particularly important when theoretical results serve as predictions for experiments. The influence of long-range corrections on polarizability in the investigated porphyrin complexes will be the subject of our future study.

## Figures and Tables

**Table 1:** Orbital exponent values for the single uncontracted f-type polarization function in the ZPol-A basis set.

Atom	Exponent
Sc	0.4120
Ti	0.5060
V	0.5740
Cr	0.0686
Mn	0.6750
Fe	0.7440
Co	0.8490
Ni	0.9560
Cu	0.8820
Zn	1.1000

**Table 2:** Nonrelativistic finite field atomic polarizabilities calculated using the ZPol and ZPol-A ^1^ basis sets. All results in au.

Atom	M_L_	ZPol [31]	ZPol-A	Ref. [34]
Sc	0	143.46	143.84	145.03
	1	146.68	147.00	148.13
	2	154.75	154.80	155.86
	average	149.26	149.49	150.68
Ti	0	125.77	126.09	127.45
	1	126.11	126.73	128.12
	2	128.36	128.67	129.48
	3	129.97	131.91	131.44
	average	127.81	128.67	129.36
V	0	112.74	112.96	114.95
	1	112.36	112.79	114.30
	2	112.04	112.30	113.19
	3	110.01	111.47	111.36
	average	111.65	112.30	113.23
Cr	0	106.26	106.78	112.88
Mn	0	89.20	89.33	90.14
Fe	0	76.64	76.82	77.62
	1	77.56	77.72	78.60
	2	80.48	80.60	81.62
	average	78.54	78.70	79.61
Co	0	69.52	69.72	70.59
	1	69.75	69.96	70.96
	2	70.50	70.69	71.49
	3	71.18	71.89	72.35
	average	70.34	70.69	71.46
Ni	0	63.99	64.13	65.39
	1	63.87	64.05	65.16
	2	63.66	63.81	64.68
	3	62.85	63.41	63.94
	average	63.54	63.81	64.71
Cu	0	73.46	73.68	77.19
Zn	0	53.22	53.37	54.07

^1^ Symbol ZPol-A denotes the basis set with the optimal single f-type function.

**Table 3:** Electric dipole moments of the first-row transition metal oxides. All results in au. Root mean square error (RMSE) values are calculated with respect to the available literature results (RMSE_1_), and to the aug-cc-pVQZ values reported in this work (RMSE_2_).

Molecule	ZPol ^1^	ZPol-A ^2^	aVQZ	Ref. ^3^
ScO	1.570	1.511	1.518	1.54
TiO	1.358	1.315	1.346	1.38
VO	1.317	1.295	1.345	1.42
CrO	1.537	1.562	1.579	1.53
MnO	2.042	2.062	2.004	1.96
FeO	2.019	2.055	1.954	–
CoO	1.854	1.886	1.999	–
NiO	1.876	1.937	1.842	–
CuO	2.065	2.109	1.949	2.01
ZnO	2.165	2.161	2.163	2.11
RMSE_1_	0.06	0.08	–	–
RMSE_2_	0.07	0.08	–	–

^1^ Reference [31]. ^2^ Original ZPol set is used on the oxygen atom. ^3^ Reference dipole moment values for ScO, TiO, VO, CrO, MnO and CuO are taken from [35], reference dipole moment values for ZnO from [36].

**Table 4:** Average static electric polarizability of the investigated porphyrin complexes calculated using the DFT/B3LYP method ^1^.

Basis set on Zn atom	ZPol	ZPol-A	SVPD	TZVPD	aVDZ	aVTZ	aVQZ
Porphyrin–zinc complex	337.85	337.67	337.02	338.33	339.15	339.24	339.96
Porphyrin–zinc–thiazole complex	382.53	382.33	382.07	383.85	385.15	385.26	386.07

^1^ Symbol aVXZ denotes the aug-cc-pVXZ (X = D, T, Q), SVPD—the def2-SVPD, and TZVPD—the def2-TZVPD basis sets. Results in au.

## Data Availability

The original contributions presented in the study are included in the article/Supplementary Material, further inquiries can be directed to the corresponding author.

## Supplementary Materials

The following supporting information can be downloaded at: https://www.mdpi.com/article/10.3390/ijms252011044/s1.
