# Supplementary material for: Theoretical Investigation of Electric Polarizability in Porphyrin–Zinc and Porphyrin–Zinc–Thiazole Complexes Using Small Property-Oriented Basis Sets
Source: Int J Mol Sci. 2024 Oct 14;25(20):11044. doi: 10.3390/ijms252011044 (PMC11507248; doi:10.3390/ijms252011044)
Supplement: Supplementary file 1 [file ijms-25-11044-s001.zip › ijms-3253227-supplementary.pdf]

Theoretical Investigation of Electric Polarizability in Porphyrin–Zinc and Porphyrin–Zinc–Thiazole Complexes Using Small Property-Oriented Basis Sets

Arkadiusz Kuziemski [1], Krzysztof Z. Łączkowski [2] and Angelika Baranowska-Łączkowska [1,*]

[1] Faculty of Physics, Kazimierz Wielki University, Powstańców Wielkopolskich 2, 85-090 Bydgoszcz, Poland
[2] Department of Chemical Technology and Pharmaceuticals, Faculty of Pharmacy, Collegium Medicum, Nicolaus Copernicus University, Jurasza 2, 85-089 Bydgoszcz, Poland
* Correspondence: anxela@ukw.edu.pl

Contents

1. **Table S1.** Optimized geometrical parameters of the porphyrin–zinc complex.
2. **Table S2.** Optimized geometrical parameters of the porphyrin–zinc–thiazole complex.

Table S1. Optimized geometrical parameters of the porphyrin–zinc complex.

Atom	x	y	z
**C**	3.041887	-0.495742	-0.000003
**C**	4.022716	-1.558747	-0.000006
**C**	3.336124	-2.735362	-0.000007
**C**	1.928117	-2.404410	-0.000002
**C**	-0.495742	-3.041886	0.000001
**C**	-1.558747	-4.022715	0.000005
**C**	-2.735362	-3.336124	0.000007
**C**	-2.404411	-1.928116	0.000002
**C**	-3.041887	0.495742	-0.000001
**C**	-4.022716	1.558747	-0.000007
**C**	-3.336124	2.735362	-0.000008
**C**	-1.928117	2.404410	-0.000002
**C**	0.495742	3.041886	0.000001
**C**	2.404410	1.928116	0.000000
**C**	2.735362	3.336124	0.000007
**C**	1.558747	4.022716	0.000007
**C**	-0.873963	3.323064	-0.000001
**C**	-3.323065	-0.873963	0.000000
**C**	0.873963	-3.323064	-0.000001
**C**	3.323065	0.873963	-0.000001
**Zn**	0.000000	-0.000001	0.000001
**N**	1.786658	-1.042571	0.000002
**N**	-1.042572	-1.786657	0.000001
**N**	1.042571	1.786658	-0.000003
**N**	-1.786658	1.042571	0.000004
**H**	5.093644	-1.416268	-0.000009
**H**	3.738949	-3.737818	-0.000010

**H**	-1.416267	-5.093643	0.000007
**H**	-3.737818	-3.738949	0.000008
**H**	-5.093644	1.416268	-0.000011
**H**	-3.738949	3.737818	-0.000012
**H**	3.737818	3.738949	0.000012
**H**	1.416267	5.093643	0.000012
**H**	-1.149773	4.371737	-0.000001
**H**	-4.371737	-1.149774	-0.000002
**H**	1.149773	-4.371736	-0.000001
**H**	4.371737	1.149775	0.000000

Table S2. Optimized geometrical parameters of the porphyrin–zinc–thiazole complex.

Atom	x	y	z
**Zn**	-0.074309	0.000000	-0.407749
**N**	-0.102231	-2.072397	-0.751782
**N**	1.971834	0.000003	-0.892419
**N**	-2.169652	-0.000002	-0.543632
**N**	-0.102236	2.072398	-0.751780
**C**	-1.205923	-2.876251	-0.719219
**C**	-0.793577	-4.259572	-0.847632
**C**	0.563219	-4.259571	-0.962757
**C**	0.991328	-2.876232	-0.905577
**C**	2.765302	-1.104147	-1.037830
**C**	4.133103	-0.681619	-1.255871
**C**	4.133100	0.681631	-1.255874
**C**	2.765299	1.104156	-1.037830
**C**	0.991320	2.876236	-0.905575
**C**	0.563208	4.259573	-0.962757
**C**	-0.793588	4.259572	-0.847625
**C**	-1.205931	2.876249	-0.719217
**C**	-2.976160	1.103854	-0.550817
**C**	-2.976157	-1.103861	-0.550818
**C**	-4.361344	-0.681443	-0.533117
**C**	-4.361346	0.681432	-0.533111
**C**	-2.527997	2.429016	-0.609584
**C**	2.313031	2.429325	-1.020287
**C**	2.313038	-2.429318	-1.020288
**C**	-2.527991	-2.429022	-0.609587
**H**	-1.460194	-5.109937	-0.859997
**H**	1.218203	-5.109948	-1.087330
**H**	4.973299	-1.343889	-1.408368
**H**	4.973294	1.343903	-1.408373
**H**	1.218189	5.109952	-1.087332
**H**	-1.460207	5.109934	-0.859987
**H**	-5.215017	-1.343971	-0.539631
**H**	-5.215021	1.343958	-0.539621
**H**	-3.295191	3.195736	-0.605621
**H**	3.069588	3.196201	-1.146819
**H**	3.069597	-3.196192	-1.146819
**H**	-3.295182	-3.195744	-0.605627

**C**	-0.498956	-0.000003	4.081769
**C**	-0.879818	-0.000002	2.776814
**S**	1.230428	-0.000002	4.192004
**N**	0.156403	-0.000001	1.869967
**C**	1.316133	0.000000	2.465169
**H**	-1.116058	-0.000004	4.965465
**H**	-1.896216	-0.000003	2.411060
**H**	2.258757	0.000001	1.936672
